# Violence Against Women in Tanzania and its Association With Health-Care Utilisation and Out-of-Pocket Payments: An Analysis of the 2015 Tanzania Demographic and Health Survey

**DOI:** 10.24248/EAHRJ-D-19-00012

**Published:** 2019-11-29

**Authors:** Seema Vyas

**Affiliations:** a Department of Population Health, London School of Hygiene and Tropical Medicine, London, UK; b Department of Epidemiology and Biostatistics, Kilimanjaro Christian Medical University College, Moshi, Tanzania

## Abstract

**Background:**

Violence against women is a major public health concern. In addition to adverse physical, mental, and sexual and reproductive health consequences, violence against women confers a considerable cost to health services and the health sector as well as to individuals and households in the form of out-of-pocket expenditures. This study aimed to assess whether physical or sexual violence against women is associated with higher health-care utilisation rates and out-of-pocket expenditures in Tanzania.

**Methods:**

This study used data from the 2015 Tanzania Demographic and Health Survey. Multivariate regression analysis was used to assess the association between health-care utilisation and partner and non-partner violence among 9,304 women. Outpatient and inpatient health expenditures were analysed using means and t-tests.

**Results:**

Women who had ever experienced physical or sexual violence (partner or non-partner) were significantly more likely to utilise health services, and in particular outpatient services, than never abused women. Out-of-pocket expenditures for out-patient care, however, did not differ by abuse status. This was in contrast to inpatient care, wherein, although abused women were not more likely to have higher utilisation rates compared with never abused women, abused women were significantly more likely to incur higher average out-of-pocket expenditures for inpatient visits. This significant difference in expenditure was possibly because of the different inpatient services sought—abused women were more likely to seek care because of illness, while never-abused women were more likely to seek care for pregnancy and delivery.

**Conclusion:**

This study highlights how violence against women in Tanzania potentially translates to higher health-care utilisation, possibly because of the long-term or chronic effects of persistent abuse. Health-care policies should, therefore, consider issues such as accessibility and affordability for health services. Additionally, governments should address the issue of violence against women more widely, thereby reducing their own costs as well.

## INTRODUCTION

The adverse health outcomes associated with violence against women have been well documented. Abused women are more likely to report poorer physical, mental and sexual and reproductive health.^1–5^ Direct health effects of violence include non-fatal injuries which range from minor ones such as bruises and abrasions, to more serious ones such as fractures and injuries to the head, face and neck.^6^ Indirect effects include health consequences caused by violence or by the acute stress of being in a violent relationship which persist even after the violence has ceased.^3,5,6^ Examples of such long-term health consequences include headaches and back pain, recurring central nervous system symptoms such as fainting and seizures, neurological sequelae, gastrointestinal problems and disorders such as loss of appetite and chronic irritable bowel syndrome, and cardiac diseases and symptoms such as hypertension and chest pain.^5,6^ In recent years, the health consequences associated with violence during pregnancy has also received attention. A meta-analysis on the rates of partner violence during pregnancy in African countries estimated an overall prevalence of 15%.^7^ Such violence has been associated with increased risk of miscarriage, abortion, antepartum haemorrhage and perinatal death.^8^

Research suggests that the utilisation of health care is greater among abused women when compared to non-abused women.^9–12^ Violence against women, therefore, confers a considerable cost to health services and the health sector.^13^

The costs of violence against women to health systems have been well documented in high income countries. For example, in the USA, the direct cost of medical and mental health-care services attributed to intimate partner rape, physical assault, and stalking was estimated to amount to US$4.1 billion (in 1995).^14^ A 1997–98 study from 2 primary care clinics in South Carolina, USA, estimated that Medicaid expenditure was slightly over US$1,000 higher among abused women when compared to the expenditure for non-abused women.^9^ In Australia, the approximate cost to the health system attributed to domestic violence was estimated to be US$1.05 billion (in 2015–2016).^15^

Typically, however, LMICs have low public expenditures on health, and gaps in expenditure to treat victims are most likely filled by out-of-pocket payments.^16,17^ Understanding the extent of out-of-pocket expenditures on health because of violence against women is important to inform policies that ensure financially a?ordable health services to abused women.

Few studies from LMIC, however, have explored the costs to households from women's utilisation of health care resulting from violence. Population-based household studies estimated that the average out-of-pocket costs related to an incident of domestic violence amounted to US$211 in Morocco and US$5.00 in Uganda.^18^ When extrapolated to national level, these expenditures amounted to almost 10% and 1.5% of per capita gross national income (GNI) in the 2 countries respectively.^18^ In Vietnam, the estimated out-of-pocket expenditure to households was 804,000 VND (US$41.2) per violent incident (in 2010), which amounted to 3.2% of per capita GNI.^19^ A study conducted in 5 health centres in Tehran, Iran, estimated that on average, victims of abuse paid US$66.8 for healthcare services (which included US$12.8 for a consultation with a physician, US$12.02 for prescribed drugs, US$16.5 for physical examinations, and US$25.5 for laboratory tests).^20^ Finally, a study in South Africa estimated that abused women paid, on average, US$265 per violent incidence for visiting a health professional (in 2008).^21^

The 2015 Tanzania Demographic Health Survey (DHS) documented that 43.6% of women had experienced physical or sexual violence by a partner since the age of 15, or physical violence by a non-partner since the age of 15, or sexual violence as a child or adult by a non-partner.^22^ Moreover, 8.1% of ever pregnant respondents reported that they had experienced physical violence during pregnancy.^22^ Among women who had experienced physical or sexual violence by a current or former partner, 70% had injuries—virtually all reported cuts bruises or aches but 15% reported serious injuries such as deep wounds and broken bones or teeth.^22^ Research in Tanzania has also documented higher levels of self-reported physical and mental ill-health symptoms, such as pain, dizziness and memory loss, among women who had ever experienced physical or sexual partner violence.^3^ In addition, a clinic-based study in Moshi found significantly higher levels of post partum depression among women who reported physical or sexual violence or emotional abuse during their pregnancy.^23^

Despite the unacceptably high prevalence of violence against women in the country, little is known about the relationship between health-seeking behaviour and out-of-pocket expenditures. This study helps to fill this knowledge gap. The overall aim of this study was to assess whether physical or sexual violence against women was associated with higher health-care utilisation rates and out-of-pocket expenditures in Tanzania. Specifically, this study explored whether women who had experienced physical or sexual violence, from either a partner or non-partner, were more likely to have used out-patient or inpatient health services; and whether aggregate out-of-pocket expenditures for seeking health care was higher among abused women when compared to never abused women.

## METHODS

This study used data from the nationally representative Tanzania DHS collected from August 2015 to February 2016. The Tanzania DHS is cross-sectional by design and used a 2-stage sampling process. In the first stage, 608 enumeration areas or “clusters” were selected from all 30 regions of the country (25 from mainland Tanzania and 5 from Zanzibar).^22^ Within each cluster, 22 households were randomly selected. The household survey was administered and completed in 12,563 (of 13,376) households. A woman's questionnaire was administered to all eligible women (ages 15-49 and resident or slept in the house the night before) in each household. A module on domestic violence was administered to 1 randomly selected woman in each household where women eligible for the woman's survey resided which yielded a sub-sample of 9,322 respondents. Interviews from 2 women from the same household were not conducted in order to guarantee privacy.

### Health-Seeking Behaviour

The household questionnaire included modules on outpatient and inpatient health seeking and expenditures. The modules asked about each household member's health seeking as an outpatient (health care that did not involve an overnight stay at the facility) in the 4 weeks to interview, and as an inpatient (health care that involved an overnight stay at a facility) in the 6 months to interview. An affirmative response deemed the household member eligible for either or both modules.

For the outpatient module, within each household, 1 eligible respondent was randomly selected to elicit further information on the following: where they had got health care from; the amount of money spent on treatment and services for the most recent visit; the reason for seeking care; additional visits to health facilities (not requiring an overnight stay) in the past 4 weeks; and other money spent on medication without consultation. Responses to the question on the reason for seeking care were categorised: (1) family planning; (2) antenatal, delivery or postnatal care; (3) malaria; (4) fever; (5) diarrhoea; (6) HIV/STDs; (7) other illness; (8) check-up/preventative; (9) accident/injury; and (10) other illnesses.

For the inpatient module, additional information was gathered from all household members who sought inpatient care in the past 6 months (up to 7 individuals in 1 household). This information included: where they got health care from; the amount of money spent on treatment and services received; and the reason for seeking health care (responses were categorised: (1) antenatal/pregnancy; (2) illness; (3) injury; or (4) other). For each household member recorded as receiving inpatient care, these questions were repeated for previous visits (up to 3 in total) in the past 6 months.

Data were analysed for respondents who reported they sought health care for reasons other than malaria (in the out-patient module) because malaria is not hypothesised to be influenced by violence in the literature. Outpatient health expenditure was measured as the amount paid for the most recent visit in the last 4 weeks—information were also collected on whether the service was free of charge. Inpatient health expenditures were aggregated across the 3 facility visits to elicit total health expenditure for inpatient care in the last 6 months.

### Violence Against Women

Women who were selected to receive the domestic violence module were asked about their experiences of physical or sexual violence by a male partner or by non-partners.

Physical or sexual partner violence against women: Ever married or cohabiting woman was asked 7 questions to illicit experience of lifetime or past year physical violence from their current/most recent partner—Did your (last) (husband/partner) ever: a) push, shake or throw something at you?; b) slap you?; c) twist your arm/pull your hair?; d) punch you with his fist?; e) kick, drag or beat you?; f) choke or burn you?; g) threaten you with a weapon?—and 3 questions on their experiences of sexual partner violence—h) physically force you to have sexual intercourse?; i) physically force you to perform other sexual acts?; j) force you with threats to perform sexual acts you did not want?

Physical Violence by a non-partner: All women who received the violence module were asked from the time they were 15 years if anyone (other than a husband/partner) had hit; slapped; kicked or done anything else to physically hurt them.

Sexual violence by a non-partner: All women who received the violence module were asked if anyone (other than a husband/partner) had ever, as a child or adult, forced them to have sexual intercourse or perform any other sexual act when they did not want.

A woman was identified as “ever abused” if she had experienced physical or sexual violence by her current, most recent or former male partner (administered to a subset of women who had ever been married or been in a cohabiting relationship); physical violence by a non-partner since the age of 15 years; or sexual violence by a non-partner either in childhood or as an adult.

### Analysis Sample

Of the 9,322 women who were selected to receive the module on domestic violence, Completed data on all forms of abuse were collected from all except 18 respondents, yielding an analysis sample of 9,304 women. Of these, outpatient health information was gathered from 600 women who had received outpatient care in the past 4 weeks and who were selected for the outpatient module. Inpatient health information was gathered from all 470 women who had received in-patient care in the past 6 months.

### Analysis

All analyses were conducted using Stata version 15.0 (StataCorp, College Station, TX, USA). Prevalence of violence and health-seeking behaviour by abused status were assessed using descriptive statistics and chi-sq test of associations, and were adjusted for clusters and sample weights.^24^ In addition, multivariate logistic regression was used to assess the association between inpatient or outpatient health care and violence adjusting for location (urban or rural, country zone; women's sociodemographic characteristics (age; partnership status; educational attainment; whether currently working; ownership of capital (house or land) assets; parity; and household socioeconomic status [SES]); and access to health care (distance from nearest health facility and whether has health insurance). Outpatient and inpatient health expenditures were analysed using means and t-test without adjusting for clusters or weights because of the low sample sizes within each cluster.

### Ethical Considerations

This study only analyses secondary publicly available data gathered as part of the DHS programme which are reviewed and approved by both international and national review boards. Additional ethical approval for this analysis was not sought. A request to access the data was made by the author and was reviewed and granted by the DHS programme. Verbal informed consent was obtained from all individual respondents included in the original study.

## RESULTS

### Prevalence of Violence Against Women

In total, 3,868 (43.2%) women reported that they had experienced physical or sexual violence by a partner or non-partner (Table 1). By violence perpetrator, the majority, 2784 (30.2%) had experienced physical or sexual violence by an intimate partner only, while a further 464 (5.0%) women experienced physical or sexual violence by both a partner and a non-partner. Six-hundred twenty women (8.0%) reported they had experienced physical or sexual violence by a non-partner only. Among women who reported they had experienced non-partner violence (n=1,084), the most commonly reported perpetrators were the respondents' immediate family members—father, mother or sibling (non-partner physical violence), for example—and acquaintances or former boyfriends (non-partner sexual violence).

**TABLE 1.** Prevalence of Violence Against Women (N=9,303)

Type of Violence	n	%
No violence	5,436	56.8
Physical or sexual partner violence	3,248	35.1
Non partner physical violence	629	8.0
Non partner sexual violence	559	6.5

### Sample Characteristics

Table 2 shows the distribution of sample characteristics. The majority, 6,461 (63.0%), of women were either married or in a cohabiting relationship, while 1,725 (23.8%) had never been married or lived with a partner. By educational attainment, 2,672 women (25.7%) had not completed primary schooling, while only 1,139 respondents (14.0%) had completed secondary education or higher. The majority, 6,902 (73.3%) had worked in the past 12 months, 2289 (44.9%) owned at least 1 type of capital asset either jointly or by themselves, and 7342 (74.6%) had at least 1 child. While 1,535 (17.2%) resided in the poorest households, 2,096 (26.9%) resided in the richest households, and 6,597 (64.0%) resided in rural areas.

**TABLE 2.** Sample Characteristics and Prevalence of Physical or Sexual Violence (N=9,303)

Characteristic	Total Sample		Physical or Sexual	*P* Value^a^
	(N=9,304)	%/Mean	%/Mean	
**Age (mean years)**	9,304	29.0	30.56	
**Partnership status**				
Never in union	1,725	23.8	21.8	<.001
Married	4,766	45.4	45.6	
Living with partner	1,695	17.6	48.2	
Widowed	242	2.9	49.3	
Divorced	444	4.6	68.3	
Separated	432	5.7	74.1	
**Educational attainment**				
No education	1,508	14.5	48.2	<.001*
Incomplete primary	1,164	11.2	50.2	
Complete primary	4,398	50.6	45.7	
Incomplete secondary	1,095	9.8	30.7	
Complete secondary or higher	1,139	14.0	31.8	
**Worked in past year**				
No	2,402	26.7	33.6	<.001
Yes	6,902	73.3	46.6	
**Ownership of assets**				
Doesn't own	5,015	55.1	35.8	<.001
Owns alone	2,213	23.6	51.2	
Joint ownership	2,076	21.3	53.2	
Parity				
No child	1,962	25.4	26.1	<.001*
1 or 2	2,982	31.9	44.7	
3 or 4	2,265	22.5	51.3	
5 or more	2,095	20.2	53.1	
**Household socioeconomic status**				
Poorest	1,535	17.2	48.5	<.001*
Poorer	1,631	17.2	46.7	
Middle	1,822	17.6	45.6	
Richer	2,220	21.1	39	
Richest	2,096	26.9	39.1	
**Location**				
Rural	6,597	64.0	44.4	.040
Urban	2,707	36.0	40.9	

a F-test with 1 degrees of freedom

Prevalence of physical or sexual violence was highest among women who were divorced or separated, women who had no secondary level education, women who reported they had worked in the past 12 months and among women who reported ownership of capital assets (sole or joint ownership). There was a significant increasing trend association between violence and parity (*P*<.001), and a significant decreasing trend associated between violence and household SES (*P*<.001).

### Health-Care Coverage and Utilisation

On average, abused women reported their nearest health facility was a distance of 3.8 km compared with a distance of 3.0 km for never abused women (*P*<.001) (Table 3). The majority, 5,966 (59.7%), reported their main method of travelling to a health facility was by walking (data not shown), a figure that varied very little by abused status (2373 (58.1%) among abused women versus 3,593 (60.9%) among never abused women). Relatively few women, 783 (9.5%) had health insurance. By abused status, 299 (8.7%) abused women had health insurance compared with 484 (9.1%) never abused women, although this result was not significant (*P*=.116).

**TABLE 3.** Health-Care Access and Utilisation by Abuse Status (N=9,303)

Variable	Never abused n=5,436 %/Mean	Abused n=3,868 %/Mean	*P* Value (Chi-square)
**Distance to nearest health facility (kilometres)**	3.03	3.79	<.001*
**Health insurance**	10.1	8.7	0.116
**Outpatient visit in past 4 weeks**	11.2	15.5	<.001
**Reason for seeking health care**			
ANC	4.4	4.2	0.053
Fever	21.3	14.6	
Diarrhoea	1.2	8.4	
HIV/STD	1.1	1.6	
Other illness	52.8	53.7	
Check-up/preventative	1.2	0.6	
Accident/injury	2.3	2.0	
Other	15.7	14.9	
**Number of times visited facility**	1.21	1.19	0.769*
**Inpatient visits in past 6 months**	5.0	5.5	0.470
**Number of inpatient visits**	273	213	
1	96.5	93.8	0.076
2	2.5	6.2	
3	1.0	0.0	
**Health facility visits**	15.6	19.6	<.001
**Overlap by type of facility visit**			
Outpatient only	10.5	14.2	<.001
In patient only	4.4	4.2	
Both	0.6	1.3	

### Outpatient Visits

Abused women were significantly more likely than never abused women to have visited a health facility as an out-patient in the last 4 weeks (n=565 (15.5%) abused women versus n=595 (11.2%) never abused women; *P*<.001) (Table 3). In both groups, the most common reasons given for seeking health care was “other illness”. Among those who visited a facility, the average number of facility visits did not differ by abused status—never abused women visited a facility on average 1.21 times and abused women visited a facility on average 1.19 times (*P*=.769).

### Inpatient Visits

There was no significant difference in the proportion of abused and never abused women who had visited a health facility as an inpatient (5.5% (n=207) abused and 5.0% (n=262) never abused; *P*=.470). Among never abused women who had been an inpatient in the past 6 months, 11 (3.5%) had been an inpatient more than once, and these figures were 11 (6.2%) among abused women (*P*=.076). In total, 493 inpatient care visits were recorded when factoring that some women received inpatient care more than once. Among never abused women, the main reasons for seeking care were because of pregnancy/delivery (n=142, 52%); illness (n=112, 41%); accident/injury (n=7, 4%) and “other reasons” (n=14, 5%) (data not shown). Among abused women, the main reasons cited were illness (n=101, 46%); pregnancy/delivery (n=89, 41%); accident/injury (n=8, 4%); and “other reasons” (n=20, 9%).

When considering the overlap of health facility visits, significantly fewer never abused women (813, 15.6%) had visited a health facility either as an outpatient or as an inpatient; a figure that is significantly lower than the 727 (19.6%) abused women who had been an inpatient or outpatient (*P*<.001). While for both groups, the majority of cases were outpatient only, 44 (0.6%) never abused women reported both outpatient and inpatient visits compared with 45 (1.3%) abused women.

Results from the regression analysis on health-care utilisation are shown in Table 4. In the unadjusted model, the odds of an inpatient or outpatient visit was 1.33 times higher among abused women (odds ratio 1.33; 95% confidence interval [CI], 1.15 to 1.53); in the adjusted model (adjusting for location; women's sociodemographic characteristics; and factors relating to health-care access), the odds of at least 1 inpatient or outpatient visit remained significantly higher among abused women (adjusted odds ratio 1.26; 95% CI, 1.08 to 1.47).

**TABLE 4.** Unadjusted and Adjusted Logistic Regression Estimates of Facility Visits (N=9,303)

	Odds Ratio	95% Confidence Interval	Adjusted Odds Ratio^a^	95% Confidence Interval
Inpatient or outpatient facility visit	1.33	1.15–1.53	1.26	1.08–1.47

† Adjusted for: urban/rural; zone; health insurance; distance from facility; age; partnership status; education; work status; ownership of capital assets; parity; household SES

### Out-of-Pocket Expenditures

#### Outpatient Expenditure

Of the 600 women who sought outpatient health care and who were selected for the outpatient module, virtually all reported that they had paid some money for the care they received—2 respondents reported that the service had been free of charge and 18 respondents did not know or remember how much was paid (Table 5). The average out-of-pocket expenditure, among those who recalled the amount of money paid (n=580), was TZS11,433 (US$5.27) and ranged from TZS200-150,000 (US$0.09–69.13). There was no significant difference in expenditure by abused status (TZS11,014 (US$5.08) never abused; TZS11,867 (US$5.47) abused; *P*=.532). In addition, there was virtually no difference in average out-of-pocket payment by type of abuse (experience of physical violence and experience of sexual violence).

**TABLE 5.** Out-of-Pocket Expenditures for Outpatient and Inpatient Care by Abuse Status

	**All Women (n=580)**		**Never Abused (n=295)**		**Abused (n=285)**			**Physical Violence (n=254)**		**Sexual Violence (n=142)**	
**Outpatient**	**TZS**	**US$**	**TZS**	**US$**	**TZS**	**US$**	***P* value**	**TZS**	**US$**	**TZS**	**US$**
Mean	11,433	5.27	11,014	5.08	11,867	5.47	0.532	11,843	5.46	11,695	5.39
Lower range	200	0.09	200	0.09	200	0.09		200	0.09	200	0.09
Upper range	150,000	69.13	95,000	43.79	150,000	69.13		150,000	69.13	150,000	69.13
	**All Women (n=340)**		**Never Abused (n=183)**		**Abused (n=157)**			**Physical Violence (n=143)**		**Sexual Violence (n=75)**	
**Inpatient**	**TZS**	**US$**	**TZS**	**US$**	**TZS**	**US$**	***P* value**	**TZS**	**US$**	**TZS**	**US$**
Mean	61,913	28.54	52,308	24.11	73,110	33.70	0.029	71,421	32.92	67,009	30.88
Lower range	2,000	0.92	2,000	0.92	3,000	1.38		3,000	1.38	3,000	1.38
Upper range	600,000	276.54	600,000	276.54	600,000	276.54		600,000	276.54	480,000	221.23

#### Inpatient Expenditure

Among the 469 respondents who sought inpatient health care, the majority, 340, (72%) paid money. In total 105 (22.6%) reported they received care free of charge while 24 (5%) did not know or remember how much money they had paid. Women who reported they had been an inpatient in the past 6 months and had paid money spent an average of TZS61,913 (US$28.54) and payment ranged from TZS2,000-600,000 (US$0.92-276.54). The out-of-pocket expenditure was significantly higher among abused women who paid an average of TZS73,110 (US$33.70), compared to TZS52,308 (US$24.11) among never abused women (*P*=.029). By type of abuse, women who experienced physical violence paid, on average, slightly more than women who had experienced sexual violence (physical violence: TZS71,400 [US$32.92]; sexual violence: TZS67,009 [US$30.88]).

## DISCUSSION

This study sought to explore the associations between violence against women and health-care utilisation and out-of-pocket payments in Tanzania using the 2015 DHS data. Prevalence of violence against women in the country is high, 43% of women, ages 15-49 years, self-reported to have experienced physical or sexual violence by either a partner or non-partner. In addition to the high prevalence of violence against women, this study found health-care utilisation (outpatient in the past 4 weeks and inpatient in the past 6 months) was significantly higher among abused women compared to never abused women— although the significant difference is because of higher utilisation of outpatient care. In addition, the majority of women who sought health care paid some money out-of-pocket.

Before discussing the implications of these findings, limitations of this study are important to highlight. Firstly, this study could not assess the costs of accessing health services directly resulting from a violent incident. Rather, this study assessed correlations between women's experiences of physical or sexual violence and health-care utilisation and expenditures. Secondly, the measure of out-of-pocket expenditures is crude and lacked disaggregation in terms of what the costs entailed. Thirdly, household self-report on individual women's use of health care and expenditures are subject to recall errors—gathering data on these aspects from health facilities could provide more reliable estimates of how much patients pay for health care.^21^

Despite these limitations this study yields insights on the implications of violence against women on the health sector and to individuals and households. Research in this area has received considerable attention in the last few years as prevention and response efforts are scaled up to address the issue. This study's finding that abused women have greater health-care utilisation is consistent with findings elsewhere.^25^ That health-care workers are trained to respond appropriately to women who have experienced violence has long been advocated for.^26^ However, studies from Tanzania, and other LMIC countries, have documented that stigma and shame prevent many women from disclosing violence to health professionals despite health worker efforts to encourage women to disclose.^25,27^ A possible implication of this is that abused women may not be receiving the range of health care to ensure their well-being, and additional strategies to address norms around violence against women and around help-seeking behaviour are needed. For example, a gender-based violence (GBV) intervention in Mbeya Region, which combined a health facility intervention with a community mobilisation programme, increased utilisation of GBV focused services.^28^ In addition, the intervention was successful in reducing community-wide tolerance of violence against women, a significant finding given consistent findings from Tanzania that gender inequitable attitudes increase women's risk of violence.^29,30^

This study also highlights that seeking health care in Tanzania requires individuals to have money at hand. Outof-pocket expenses are reported to prevent many women from seeking health services immediately after a violent incident, or are associated with catastrophic health expenditure.^27,31–34^ This barrier to seeking health care may be further exacerbated when taking into consideration the lost earnings to women because of violence. For example, research in Tanzania suggests that women who experienced physical or sexual violence by their male partner (in the past year) earned 35% less than their non-abused counterparts.^35^ The evidence base from LMIC on mechanisms to address user fees and improving quality of clinical care to increase uptake of GBV services, however, is limited.^36^ An intervention in Kenya involved distributing vouchers offering GBV recovery care free of charge to women attending reproductive health facilities. Although a full-scale intervention evaluation was not undertaken, qualitative research identified reservations by community members on the validity of the vouchers and also on shame and stigma in accessing targeted GBV care.^37^

## CONCLUSION

In summary, violence against women is likely to confer a cost onto an already under resourced health system, and also onto individuals and households for treatment. Health-care policies should, therefore, consider issues such as accessibility and affordability for health services and also addressing the barriers of stigma and shame in reporting experience of violence. In addition, governments should address the issue of violence against women more widely, thereby reducing their own costs as well.
